# Novel Variants in the SLC16A2 Gene Associated With Allan–Herndon–Dudley Syndrome in China

**DOI:** 10.1155/humu/3319748

**Published:** 2026-05-16

**Authors:** Wei Li, Zijia Sun, Xiao Wu, Fen Lu, Qiaoli Zhou, Xiaoqin Zhang, Min Zhu

**Affiliations:** ^1^ Department of Rehabilitation, Children′s Hospital of Nanjing Medical University, College of Pediatrics, Nanjing Medical University, Nanjing, Jiangsu, China, njmu.edu.cn; ^2^ Department of Endocrinology, Children′s Hospital of Nanjing Medical University, College of Pediatrics, Nanjing Medical University, Nanjing, Jiangsu, China, njmu.edu.cn; ^3^ Department of Outpatient, Children′s Hospital of Nanjing Medical University, Nanjing, Jiangsu, China

**Keywords:** Allan–Herndon–Dudley syndrome, monocarboxylate Transporter 8, *SLC16A2* gene, thyroid hormone

## Abstract

**Objective:**

This study is aimed at investigating the genetic defects and clinical features of Chinese children with *SLC16A2* variants and at exploring the effects of mutant MCT8 on protein expression and subcellular localization through in vitro experiments.

**Methods:**

Children with intellectual disability and abnormal serum thyroid hormone levels were screened using whole‐exome sequencing (WES). We collected patients′ clinical data and assessed their cognitive, linguistic, and motor abilities. Candidate variants were verified by Sanger sequencing, and their pathogenicity and evolutionary conservation were analyzed using in silico prediction tools. Protein expression and subcellular localization of mutant MCT8 were evaluated by Western blotting and immunofluorescence microscopy.

**Results:**

Exome sequencing identified seven previously uncharacterized *SLC16A2* variants in 10 unrelated male patients (nine families). These included three missense mutations (p.Ala150Thr,p.Gly208Arg, and p.Gly208Asp), three frameshift mutations (p.Leu168fs, p.Ser243Cysfs, and p.Val485fs), one deletion–insertion mutation (p.Ile328_Ala329delinsThr), and one balanced translocation, t(X,12) (q13.2; q13.13). All patients exhibited global developmental delay, axial hypotonia, dystonia, and abnormal thyroid hormone profiles. In vitro experiments demonstrated significantly reduced expression of mutant proteins compared with the wild‐type. Immunofluorescence showed that, in addition to residual plasma membrane localization, mutant proteins were also partially retained in the cytoplasm, whereas the wild‐type protein localized exclusively to the plasma membrane.

**Conclusion:**

Our findings expand the genotypic and phenotypic spectrum of MCT8 deficiency. The results suggest that *SLC16A2* variants lead to a loss of function through decreased protein expression and defective plasma membrane trafficking.

## 1. Introduction

Thyroid hormone (TH) transporters facilitate the transmembrane transport of thyroxine (T4) and triiodothyronine (T3), which is essential for normal TH metabolism and signaling. Among the numerous TH transporters identified, the most specific is monocarboxylate Transporter 8 (MCT8), encoded by the X‐linked *SLC16A2* gene (OMIM 300095) [1]. MCT8 is widely expressed in humans, with high levels observed in the brain, liver, kidney, and thyroid [2].

Mutations in the *SLC16A2* gene cause MCT8 deficiency, an X‐linked disorder known as Allan–Herndon–Dudley syndrome (AHDS) (OMIM 300523) [3]. The condition predominantly affects males, who typically present with severe psychomotor retardation and a characteristic thyroid phenotype: elevated serum T3, low T4, and normal TSH. Although traditionally considered an X‐linked recessive disorder limited to males, a recent landmark study by Groeneweg et al. demonstrated that female heterozygous carriers with skewed X‐chromosome inactivation can also manifest variable neurocognitive impairment, behavioral abnormalities, and abnormal TH profiles [4]. MCT8 is critical for TH transport across the blood–brain barrier and plays an essential role in both fetal and postnatal neurodevelopment. Impaired central MCT8 function is widely accepted to result in cerebral hypothyroidism, leading to neurological damage and psychomotor deficits [5]. Peripherally, altered TH secretion and increased conversion of T4–T3 contribute to mild peripheral thyrotoxic features, such as low body weight and tachycardia. Importantly, brain injury in MCT8 deficiency begins in utero [6]. Prenatal administration of TH analogues that cross the placenta and are concentrated by the fetus—such as DITPA—has been shown to improve neuromotor and cognitive outcomes in affected children. Collectively, these findings support the potential value of TH screening in the neonatal or even fetal period to enable early intervention and improve long‐term prognosis.

To date, over 100 different mutations in the *SLC16A2* gene have been reported, including missense, nonsense, deletion, insertion, splicing, and complex rearrangements. These mutations variably impair MCT8‐mediated TH transport and may involve diverse pathogenic mechanisms, with many affecting protein expression and subcellular localization [7].

In this study, we identified 10 unrelated patients (nine families) with *SLC16A2* mutations from a cohort of children with intellectual disability, movement disorders, and TH abnormalities using WES. We summarize their clinical phenotypes and diagnostic processes, and functionally characterize two novel *SLC16A2* mutations by assessing protein expression and subcellular localization in vitro.

## 2. Materials and Methods

### 2.1. Patients and Ethical Considerations

Ten patients with *SLC16A2* variants were identified by WES among individuals presenting with psychomotor retardation and abnormal serum TH levels. Sequencing results from the probands were compared with public genomic databases. Variants were classified according to the American College of Medical Genetics and Genomics (ACMG) guidelines and verified by Sanger sequencing. The study was approved by the Institutional Review Board (IRB) of the Children′s Hospital of Nanjing Medical University (Approval No. 202410006‐1). Written informed consent was obtained from the parents or guardians of all participants.

### 2.2. Plasmid Construction

Wild‐type and mutant *SLC16A2* constructs—pcDNA‐*SLC16A2*‐WT‐3xFLAG, pcDNA‐*SLC16A2*‐p.Ala150Thr‐3xFLAG, and pcDNA‐*SLC16A2*‐p.Ile328_Ala329delinsThr‐3xFLAG—were designed and synthesized by Nanjing Jinbeizin Biotechnology Co., Ltd. All constructs were verified by bidirectional Sanger sequencing.

### 2.3. Cell Culture and Transfection

HEK‐293T cells (human embryonic kidney cells) were cultured and transiently transfected with wild‐type or mutant *SLC16A2* plasmids as previously described [6]. Briefly, cells were seeded in six‐well plates and maintained at 37°C under 5% CO_2_. At 70%–80% confluence, cells were transfected with 2 *μ*g of purified plasmid DNA using PolyJet transfection reagent (SignaGen) according to the manufacturer′s protocol. The final plasmid concentration was adjusted to 0.01 *μ*g/*μ*L. Transfection proceeded for 4–6 h, and cells were harvested 24 h posttransfection for subsequent analysis.

### 2.4. Western Blotting

HEK‐293T cells were washed with ice‐cold PBS and lysed in RIPA buffer supplemented with protease and phosphatase inhibitors [8–10]. Lysates were sonicated briefly and centrifuged at 12,000 × g for 20 min to remove debris. Total protein concentration was determined using a BCA Protein Assay Kit. Proteins (20 *μ*g per lane) were separated by 10% SDS‐PAGE and transferred to PVDF membranes. Membranes were blocked with QuickBlock Western Blocking Solution for 15 min at room temperature and then incubated overnight at 4°C with primary antibodies against MCT8 (1:1,500; ProTech) and GAPDH (1:3,000; ProTech). After washing, membranes were incubated with HRP‐conjugated secondary antibodies for 1 h at room temperature. Protein bands were visualized by chemiluminescence, and band intensities were quantified using ImageJ software (National Institutes of Health, United States).

### 2.5. Immunofluorescence

HEK‐293T cells were seeded on glass coverslips in 12‐well plates and cultured in DMEM supplemented with 10% fetal bovine serum at 37°C and 5% CO_2_ [8, 9]. Transfection was performed as described above. Twenty‐four hours posttransfection, cells were fixed with 4% paraformaldehyde, permeabilized with 0.1% Triton X‐100, and blocked with 5% BSA. Cells were incubated overnight at 4°C with rabbit anti‐MCT8 antibody (1:400; Abcam) and mouse anti–ZO‐1 antibody (1:400; proteintech), followed by incubation with Alexa Fluor–conjugated secondary antibodies at 37°C for 1 h. Nuclei were counterstained with DAPI. Images were acquired using a confocal laser scanning microscope with a 40× objective. Excitation wavelengths were 493 nm (AF488), 633 nm (AF647), and 340 nm (DAPI).

### 2.6. Structural Modeling

The three‐dimensional structure of wild‐type human MCT8 was retrieved from the AlphaFold Protein Structure Database [11]. Models of mutant MCT8 structures were generated using SWISS‐MODEL (https://swissmodel.expasy.org/interactive).

## 3. Results

### 3.1. Clinical Features of the Probands

We identified 10 previously unreported *SLC16A2* mutations in 10 male patients aged 5 months–13 months (Table 1) (https://hpo.jax.org/) The p.Ala150Thr variant was found in both P1 and P2. Most patients were born at term with uneventful prenatal and neonatal courses, except for P1, P2, P8, P9, and P10. P1 was born to a mother with preeclampsia, and delivery was delayed by 1 week; P2 experienced perinatal hypoxia due to cord entanglement but did not require resuscitation; P8 was small for gestational age. Six patients had neonatal jaundice, three requiring therapeutic intervention. P9 and P10 were twins, conceived through in vitro fertilization. P9 was the eldest of the twins, 37 weeks gestation, delivered by cesarean section, birth weight was 2.5 kg with the history of neonatal asphyxia requiring resuscitation, neonatal pneumonia, coagulation disorder, and patent ductus arteriosus. P10 was the younger twin brother, birth weight was 2.3 kg, with the history of neonatal asphyxia requiring resuscitation, small‐for‐gestational‐age infant, neonatal pulmonary edema, neonatal infection, and patent ductus arteriosus.

**Table 1:** Clinical characteristics of patients with AHDS syndrome in China and a previous study.

Characteristic	This Cohort (*n* = 10)	P1	P2	P3	P4	P5	P6	P7	P8	P9	P10	Literature cohort^a^
**Demographics**												
Age at onset (months)	4.5 (3–10)	9	10	5	5	5	3	4	3	4	4	N/A
Age at assessment (years)	0.61 (0.41–1.06)	0.73	1.06	0.49	0.73	0.65	0.73	0.41	0.57	0.5	0.5	4.8 (0.4–66.8)

**Pregnancy and birth**												
Premature birth	0 (0%)	No	No	No	No	No	No	No	No	No	No	0.70%
Abnormal prenatal findings^b^	4 (40%)	Yes	No	No	No	No	No	No	Yes^2^	Yes	Yes	N/A

**Growth**												
Short stature	0 (0%)	Absent	Absent	Absent	Absent	Absent	Absent	Absent	Absent	Absent	Absent	27 (40.3%)
Failure to thrive in infancy	10 (100%)	Present	Present	Present	Present	Present	Present	Present	Present	Present	Present	N/A
Small for gestational age	1 (10%)	Absent	Absent	Absent	Absent	Absent	Absent	Absent	Absent	Absent	Present	N/A

**Head and neck**												
Narrow face	9 (90%)	Present	Present	Present	Present	Present	Present	Present	Absent	Present	Present	N/A
Long face	9 (90%)	Present	Present	Present	Present	Present	Present	Present	Absent	Present	Present	N/A
Myopathic facies	4 (40%)	Present	Present	Absent	Absent	Present	Present	Absent	Absent	Absent	Absent	N/A
Nystagmus	0 (0%)	Absent	Absent	Absent	Absent	Absent	Absent	Absent	Absent	Absent	Absent	26.5% (13/49)

**Cardiovascular**												
Hypertension	0 (0%)	Absent	Absent	Absent	Absent	Absent	Absent	Absent	Absent	Absent	Absent	53.2% (25/47)
Tachycardia	0 (0%)	Absent	Absent	Absent	Absent	Absent	Absent	Absent	Absent	Absent	Absent	31.3% (20/64)

**Digestive and genitourinary system**												
Feeding difficulties in infancy	5 (50%)	Present	Present	Absent	Present	Absent	Absent	Present	Present	Absent	Absent	71.4% (55/77)
Prolonged neonatal jaundice	4 (40%)	Present	Present	Absent	Absent	Present	Absent	Present	Absent	Absent	Absent	N/A
Cryptorchidism	1 (10%)	Absent	Absent	Absent	Absent	Absent	Absent	Absent	Present	Absent	Absent	18.4% (9/49)

**Endocrine and immunology**												
Abnormality of thyroid physiology	10 (100%)	Present	Present	Present	Present	Present	Present	Present	Present	Present	Present	88.7% (94/106)
Increased circulating free T3	10 (100%)	Present	Present	Present	Present	Present	Present	Present	Present	Present	Present	95.1% (96/106)
Recurrent respiratory infections	9 (90%)	Present	Present	Present	Present	Present	Present	Absen	Present	Present	Present	69% (29/42)

**Skeletal and musculature system**												
Axial hypotonia	10 (100%)	Present	Present	Present	Present	Present	Present	Present	Present	Present	Present	N/A
Spastic tetraplegia	0 (0%)	Absent	Absent	Absent	Absent	Absent	Absent	Absent	Absent	Absent	Absent	N/A
Skeletal muscle atrophy	0 (0%)	Absent	Absent	Absent	Absent	Absent	Absent	Absent	Absent	Absent	Absent	N/A
Neonatal hypotonia	1 (10%)	Absent	Absent	Absent	Absent	Absent	Absent	Absent	Present	Absent	Absent	N/A
Generalized muscle weakness	0 (0%)	Absent	Absent	Absent	Absent	Absent	Absent	Absent	Absent	Absent	Absent	N/A
Spasticity	6 (60%)	Absent	Absent	Absent	Absent	Present	Present	Present	Present	Present	Present	80.3% (57/71)
Limb hypertonia	1 (10%)	Absent	Absent	Absent	Absent	Absent	Absent	Absent	Present	Absent	Absent	N/A
Pes valgus	8 (80%)	Present	Present	Present	Present	Present	Present	Present	Present	Absent	Absent	N/A
Pes planus	8 (80%)	Present	Present	Present	Present	Present	Present	Present	Present	Absent	Absent	N/A
Ankle clonus	0 (0%)	Absent	Absent	Absent	Absent	Absent	Absent	Absent	Absent	Absent	Absent	N/A
Flexion contracture	0 (0%)	Absent	Absent	Absent	Absent	Absent	Absent	Absent	Absent	Absent	Absent	N/A
Hyperhidrosis	2 (20%)	Present	Present	Absent	Absent	Absent	Absent	Absent	Absent	Absent	Absent	N/A

**Anthropometrics (at assessment)**												
Low weight (*Z*‐score< −2)^b^	6 (60%)	No	No	Yes	Yes	Yes	N/A	Yes	Yes	No	Yes	71.1% (59/83)
Microcephaly (HC < P3)^d^	2 (20%)	N/A	N/A	N/A	Yes	Yes	No	N/A	N/A	No	No	32% (19/59)

**Neurological development**												
Poor head control	10 (100%)	Present	Present	Present	Present	Present	Present	Present	Present	Present	Present	75.3% (58/77)
Delayed speech and language development	10 (100%)	Present	Present	Present	Present	Present	Present	Present	Present	Present	Present	6.6% (5/76)
Independent sitting	0 (0%)	Absent	Absent	Absent	Absent	Absent	Absent	Absent	Absent	Absent	Absent	7.7% (6/78)
Delayed ability to walk	10 (100%)	Present	Present	Present	Present	Present	Present	Present	Present	Present	Present	100% (7/77)

**Neurological signs**												
Sleep disturbance	3 (30%)	Absent	Absent	Absent	Absent	Absent	Absent	Absent	Absent	Present	Present	39.2% (20/51)
Abnormal pyramidal sign	10 (100%)	Present	Present	Present	Present	Present	Present	Present	Present	Present	Present	25% (7/28)
Brisk reflexes	2 (20%)	Absent	Absent	Absent	Absent	Absent	Absent	Absent	Absent	Present	Present	N/A
Dystonia	10 (100%)	Present	Present	Present	Present	Present	Present	Present	Present	Present	Present	82.6% (57/69)
Hypokinesia	10 (100%)	Present	Present	Present	Present	Present	Present	Present	Present	Present	Present	N/A
Hyperreflexia	10 (100%)	Present	Present	Present	Present	Present	Present	Present	Present	Present	Present	N/A
Dyskinesia	10 (100%)	Present	Present	Present	Present	Present	Present	Present	Present	Present	Present	N/A
Seizure	0 (0%)	Absent	Absent	Absent	Absent	Absent	Absent	Absent	Absent	Absent	Absent	23.1% (15/65)
Persistent primitive reflexes	9 (90%)	Present	Absent	Present	Present	Present	Present	Present	Present	Present	Present	91.1% (51/56)
Intellectual disability	10 (100%)	Present	Present	Present	Present	Present	Present	Present	Present	Present	Present	N/A
Babinski sign^e^	6 (60%)	Present	Absent	Absent	Absent	Present	N/A	Present	Present	Present	Present	66.7% (38/57)

**Brain MRI findings**												
Widened extracerebral space (WECS)	10 (100%)	Present	Present	Present	Present	Present	Present	Present	Present	Present	Present	100% (13/13)
Delayed myelination (DM)	10 (100%)	Present	Present	Present	Present	Present	Present	Present	Present	Present	Present	100% (13/13)

Abbreviations: AHDS, Allan–Herndon–Dudley syndrome; N/A, not available or not assessed; P3, 3rd percentile.

^a^Literature data were consolidated from Frints et al. (PMID: 36458135) and van Geel et al. (PMID: 32559475). Values represent reported medians (ranges) or proportions.

^b^Abnormal prenatal findings: P1 had a history of fetal preservation; P8 was noted to have a small fetal size. N/A indicates data not available. P9 and P10 are twins and have abnormal prenatal history.

^c^Low weight: Defined as weight − for − age *Z* − score < −2. Data were available for 7/8 patients (P6 missing).

^d^Microcephaly: Defined as head circumference (HC) below the 3rd percentile (P3). Data were available for 5/8 patients (P1, P2, P3, P7 missing).

^e^Positive Babinski sign: Data were available for 7/8 patients (P6 missing).

**Figure 1:**
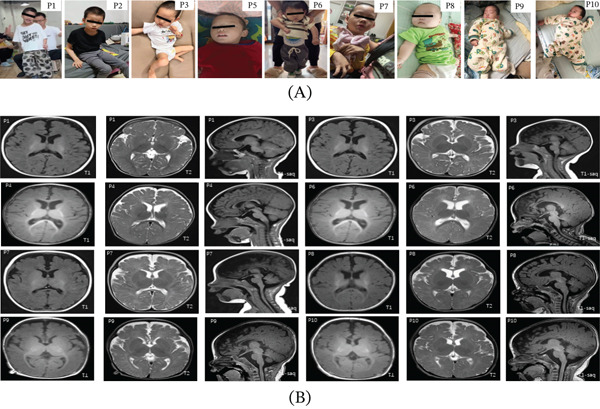
(A) Facial dysmorphisms of representative patients. Patients P1, P2, P3, P5, P6, P7, P8, P9, and P10 exhibit characteristic features including myopathic facies, long face, pale eyebrows, strabismus, large ears, and open mouth. (B) Cranial magnetic resonance imaging (MRI) findings. Images from Patients P1, P3, P4, P6, P7, P8, P9, and P10 are shown (T1‐weighted, T2‐weighted, and T1‐weighted sagittal views). Delayed myelination was observed in all patients. Widening of extracerebral spaces was present in P3, P4, P6, P7, P8, P9, and P10. Thinning of the corpus callosum was noted in P1, P4, P9, and P10.Widened and deepened cerebellar sulci was observed in P9. P1 (20 months) additionally showed delayed myelination, a thin corpus callosum, and intact bilateral ventricular morphology. All images are representative of findings at the time of diagnosis. Scale bars and MRI sequence parameters are provided in the corresponding original radiology reports.

All patients presented before 1 year of age with global developmental delay. Initial complaints included poor head control, without evidence of developmental regression. P4 and P5 exhibited low body weight and microcephaly. P2, P9, and P10 displayed choreoathetosis, initially diagnosed as dyskinetic cerebral palsy. Primitive reflexes were observed in all patients, and nine had a positive Babinski sign. Common observations included abnormal posturing (hand clenching, inwardly clasped thumbs, forearm rotation, and pes valgus) and distinctive facial features (myopathic facies, long face, pale eyebrows, strabismus, large ears, and open mouth) (Figure 1A). Eight patients had feeding difficulties and muscular hypotonia. Recurrent respiratory infections were common during hospitalization; only P7 presented with gastrointestinal infection.

Thyroid function tests consistently showed elevated free T3 (fT3), low free T4 (fT4), and normal TSH levels across all patients (Table 2). All patients exhibited markedly elevated fT3/fT4 ratios, with age‐ and sex‐specific SDS values ranging from 5.13 to 8.21, far exceeding the +3.0 upper limit of the normative reference tool. Most patients had low serum creatinine and elevated creatine kinase isoenzymes. Tachycardia was not observed. Cranial MRI revealed delayed myelination and widening of frontal and/or temporal extracerebral spaces in most patients (Figure 1B). P1 initially had a normal MRI, but follow‐up at 20 months showed delayed T2‐weighted myelination, a thin corpus callosum, and full lateral ventricles.

**Table 2:** Clinical and biochemical parameters representing peripheral features in our patients with MCT8 deficiency; RI means respiratory infection; GS means gastrointestinal infection.

Proband number	1	2	3	4	5	6	7	8	9	10
Age at assessment (months)	9	13	6	9	8	9	5	7	6	6
TSH (uIU/mL)	4.18 (normal)	NA	4.06 (normal)	6.17 (normal)	6.89 (normal)	2.84 (normal)	5.5 (normal)	4.89 (normal)	2.7 (normal)	4.67 (normal)
FT4 (pmol/L)	9.88 (↓)	NA	7.67 (↓)	9.55 (↓)	7.52 (↓)	7 (↓)	6.65 (↓)	7.13 (↓)	6.4 (↓)	7.5 (↓)
T4 (nol/L)	65.36 (↓)	NA	66.33 (↓)	64.37 (↓)	44.59 (↓)	NA	42.35 (↓)	35.9 (↓)	72.50 (↓)	77.80 (↓)
FT3 (pmo/L)	9.56 (↑)	NA	13.09 (↑)	14.73 (↑)	10.72 (↑)	6.4 (↑)	13.03 (↑)	11.4 (↑)	17.1 (↑)	14.0 (↑)
T3 (nmol/L)	4.07 (↑)	NA	6.09 (↑)	5.82 (normal)	4.09 (↑)	NA	4.57 (↑)	3.88 (normal)	7.26 (↑)	6.52 (↑)
T3/T4	0.062	NA	0.092	0.090	0.092	NA	0.108	0.108	0.100	0.084
FT3/FT4 ratio	0.97	NA	1.71	1.54	1.43	0.91	1.96	1.60	2.67	1.232
FT3_FT4_RATIO_SDS^A^	5.46	NA	> +3.0	8.13	7.63	5.13	> +3.0	8.21	> +3.0	> +3.0
RHR (bpm)	110	120	112	118	121	NA	110	NA	126	128
SBP/DBP (mmHg)	77/52	96/57	81/43	93/67	86/54	NA	86/52	NA	80/45	78/45
Creatinine (umol/L)	↓	NA	↓	↓	N	NA	↓	NA	13	Normal
CK (U/L)	N	NA	N	N	N	NA	N	NA	77	102
CK‐MB (U/L)	↑	NA	↑	↑	↑	NA	↑	NA	28	29
Triglyceride (mmol/L)	↓	NA	↑	Normal	Normal	NA	Normal	NA	0.65	0.61
Infections	RI	RI	RI	RI	RI	RI	GI	RI	RI	RI

Abbreviations: CK, creatine kinase; CK‐MB, creatine kinase isoenzymes; DBP, diastolic blood pressure; GI, gastrointestinal infection; NA, not recorded; RHR, resting heart rate; RI, respiratory infection; SBP, systolic blood pressure; TSH, thyroid‐stimulating hormone.

^A^fT3/fT4 ratio SDS: All patients had fT3/fT4 ratio SDS values exceeding the reference tool′s computational limit (+3.0), with actual raw values ranging from 5.13 to 8.21. (Source: http://www.thyroid-hormone-ratio.org/).

### 3.2. *SLC16A2* Variants

**Figure 2:**
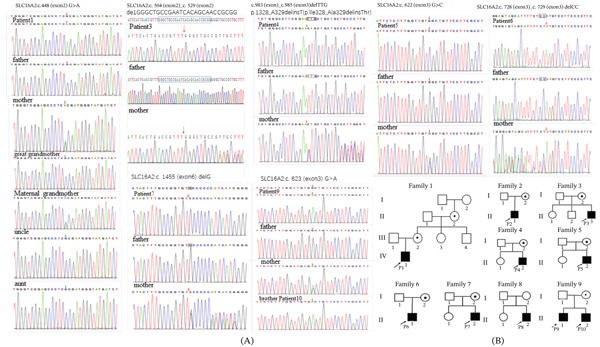
Genetic bioinformatics analysis. (A) Sanger sequencing chromatograms of P1, P3, P4, P5, P6, P7, P9, and P10 with SLC16A2 gene variants. The probands show a nucleotide change (red arrow). (B) Pedigree maps of nine unrelated families (10 patients) harboring pathogenic variants in the SLC16A2 gene; the arrows indicate the proband.

**Figure 3:**
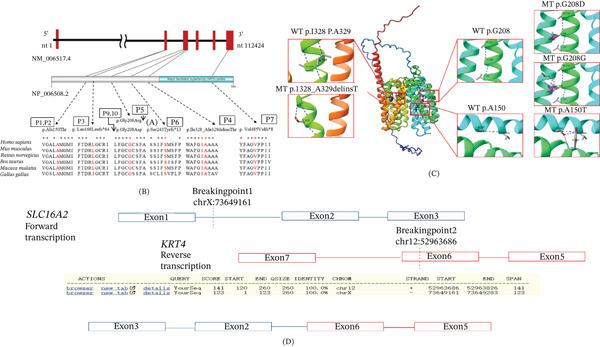
(A) Schematic representation of the SLC16A2 gene and MCT8 protein. The exon structure of SLC16A2 gene corresponds to the transcript NM_006517.4, which consists of six coding exons with 112,424 bps. Red square represents exon. The figure also illustrates the locations of the SLC16A2 gene variants identified in nine patients with AHDS. Protein domain composition of MCT8. Schematic representation of the MCT8 protein, in which rectangles denote the distinct functional domains: Blue denotes the major facilitator superfamily (MFS). (B) Multiple sequence alignment of the MCT8 protein region flanking residue Ala150, Leu168, Gly208, Ser243, Ile328_Ala, and Val485. All mutated amino acids are conserved from *Homo sapiens* to *Danio rerio*. (C) Localization of the four hMCT8 mutation and the amino acid changes. The full model structure is WT‐hMCT8. (D) The X; 12 translocation. Through whole‐exome sequencing, a translocation was identified on Chromosome 12 and the X chromosome, spanning the SLC16A2 gene. The data were verified by IGV, revealing two distinct breakpoints, both located within intronic regions. After alignment, X is the reverse sequence, and 12 is the forward sequence. This means that the sequence after Breakpoint 1 is connected in front of Breakpoint 2. It is speculated that X is fused onto chr12, as shown in the figure: The SLC16A2 gene is connected downstream of the KRT4 gene, utilizing the transcription factors and promoter of the KRT4 gene

Genetic analysis by WES and Sanger sequencing identified two missense (p.Ala150Thr, p.Gly208Arg, and p.Gly208Asp), three frameshift (p.Leu168fs, p.Ser243Cysfs, and p.Val485fs), one deletion–insertion (p.Ile328_Ala329delinsThr), and one balanced translocation, t(X; 12) (q13.2; q13.13) (Figure 2A). Three variants—p.Ala150Thr, p.Ile328_Ala329delinsThr, and t(X; 12) (q13.2; q13.13)—were novel. All probands inherited the mutation from their mothers, consistent with X‐linked recessive inheritance, except for P8, who had a de novo translocation (Figure 2B). All mutated amino acids (the MCT8 protein region flanking residue Ala150, Leu168,Gly208,Ser243,Ile328_Ala, and Val485) are conserved across multiple species (Figure 3A,B). According to ACMG guidelines, p.Ala150Thr and t(X; 12) were classified as likely pathogenic, whereas p.Ile328_Ala329delinsThr was classified as a variant of uncertain significance (Table 3). None of the novel variants were present in public genomic databases. All affected residues are highly conserved across mammalian species.IGV analysis of P8 confirmed two intronic breakpoints disrupting the *SLC16A2* gene (Figure 3D).

**Table 3:** The variants of our patients. LP means likely pathogenic; VUS means variant of uncertain significance.

Patient	Exon	Nucleotide change	Amino acid change	Mutation type	gnomAD	PolyPhen2_HDIV	PolyPhen2_HVAR	SIFT	Mutation taster	CADD	M‐CAP	ACMG classfication
P1\P2	1	c.448(exon2) G > A	p. Ala150Thr	Missense mutation	0	Benign (0.157)	Benign (0.132)	0.32	Polymorphism (0.37)	Deleterious (22)	Damaging (0.54377)	VUS: PM1 + PM2.
P3	2	c.504(exon2)c.529(exon2)delGGGCTGCCGAATCACAGCAACCGCGG	p. Leu1 68Leufs∗64	Frameshift mutation	Unrecorded	NA	NA	NA	Disease‐causing	NA	NA	LP: PVS1 + PM2.
P4	3	c.983(exon3)_c.985(exon3)del TTG	p. Ile328_A la329delinsThr	Codon mutation	Unrecorded	NA	NA	NA	Disease‐causing	NA	NA	VUS: PM2 + PM4.
P5	3	c.622(exon3) G > C	p. Gly208Arg	Missense mutation	Unrecorded	Probably damaging (1)	Probably damaging (1.0)	0	Disease‐causing	NA	Damaging (0.909387)	VUS: PM2_Supporting + PP3.
P9\P10	3	c.623(exon3)G > A	p.Gly208Asp	Missense mutation	Unrecorded	Probably damaging (1.0)	Probably damaging (1.0)	NA	Disease‐causing	Deleterious (286)	Damaging (0.883363)	VUS: PM1 + PM2_Supporting + PP3_Moderate.
P6	3	c.728(exon3)_c.729(exon3)del CC	p. Ser243fs Ter 13	Frameshift mutation	Unrecorded	NA	NA	NA	Disease‐causing	NA	NA	LP: PVS1 + PM2_Supporting.
P7	6	c.1455(exon6) delG	p. Val485Valfs∗8	Frameshift mutation	Unrecorded	NA	NA	NA	Disease‐causing	NA	NA	Pathogenic: PVS1 − S + PM2 + PP4.
P8	—	t(X; 12) (q13.2; q13.13)	p.?	Gene breakage mutation caused by balanced chromosomal translocation	Unrecorded	NA	NA	NA		NA	NA	VUS.

*Note*: This patient has a balanced chromosomal translocation, and the specific amino acid change resulting from the mutation cannot be determined, so a question mark is used to denote this.

Abbreviations: LP, likely pathogenic; VUS, variants of uncertain significance.

### 3.3. Structural Modeling

Structural models of wild‐type and mutant MCT8 were generated to assess conformational changes (Figure 3C). The p.Ile328_Ala329delinsThr variant exhibited alterations in helix formation, side‐chain orientation, and hydrogen bonding. The p.Ala150Thr variant showed changes in side‐chain conformation and hydrogen bonding compared with the wild‐type structure.

### 3.4. In Vitro Functional Characterization

**Figure 4:**
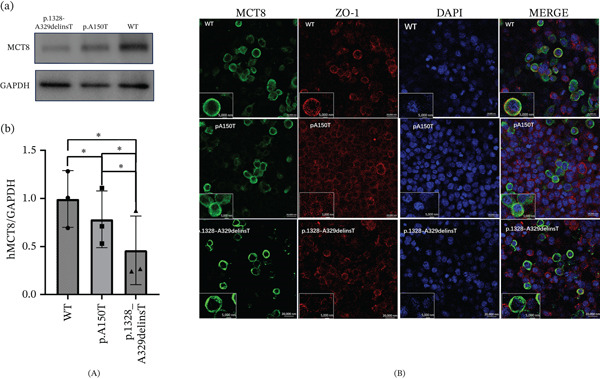
Functional characterization of wild‐type and mutant MCT8. (A) Western blot analysis of MCT8 protein expression in HEK‐293T cells transfected with wild‐type (WT) or mutant (p.Ala150Thr and p.Ile328_Ala329delinsThr) SLC16A2 constructs. GAPDH served as a loading control. Each dot represents an independent experiment (*n* = 3); horizontal lines indicate the mean, and error bars denote SD. Statistical significance was determined by unpaired *t*‐test ( ^∗∗^ *p* < 0.01,  ^∗∗∗^ *p* < 0.001). (B) Subcellular localization of wild‐type and mutant MCT8. HEK‐293T cells expressing WT or mutant MCT8 were immunostained for MCT8 (green) and the plasma membrane marker ZO‐1 (red); nuclei were counterstained with DAPI (blue). Confocal images were acquired at 20× (upper panels, scale bar = 20 *μ*m) and 100× (lower panels, scale bar = 5 *μ*m) magnification. WT MCT8 exhibits robust colocalization with ZO‐1 at the plasma membrane. In contrast, both mutant proteins show markedly reduced membrane localization and increased cytoplasmic retention, indicating impaired membrane trafficking. Images are representative of three independent experiments.

Western blot analysis of HEK‐293T cells transfected with wild‐type or mutant (p.Ala150Thr, and p.Ile328_Ala329delinsThr). *SLC16A2* constructs showed a specific band at ~60 kDa. Both mutants exhibited significantly reduced protein expression compared with the wild‐type (Figure 4A). Immunofluorescence demonstrated altered subcellular localization: Although wild‐type MCT8 localized predominantly to the plasma membrane, both mutants exhibited partial membrane localization along with markedly increased cytoplasmic retention (Figure 4B).

## 4. Discussion

AHDS, or MCT8 deficiency, is a neurodevelopmental disorder caused by defects in the MCT8 protein, leading to severe intellectual and motor disability and abnormal thyroid function [2, 12]. *SLC16A2* is the only known gene associated with AHDS. As an X‐linked condition, loss‐of‐function mutations typically cause severe manifestations in males. Females with heterozygous SLC16A2 mutations and skewed X‐chromosome inactivation exhibit a highly variable phenotype, ranging from mild learning difficulties and attention deficits to moderate‐to‐severe cognitive impairment, but generally lack the severe motor disability characteristic of affected males [4, 12]. Over 100 pathogenic *SLC16A2* variants have been reported worldwide. Here, we describe 10 Chinese probands from unrelated nine families carrying seven different *SLC16A2* mutations, including three novel ones: c.448G > A (p.Ala150Thr), c.983_985delTTGinsAC (p.Ile328_Ala329delinsThr), and t(X; 12) (q13.2; q13.13). We investigated the functional impact of two novel missense and deletion–insertion mutations, demonstrating reduced protein expression and mislocalization.

Consistent with previous reports, all patients exhibited global developmental delay, motor deficits, and recurrent infections—primarily respiratory [12, 13]. None showed developmental regression. All patients were diagnosed before age three, initially evaluated in pediatric health, endocrinology, or rehabilitation departments. Early diagnoses included global developmental delay, cerebral palsy, and thyroid dysfunction. Certain clinical features reported in other cohorts—such as epilepsy, scoliosis, incontinence, hip dislocation, nystagmus, and seizures—were not observed in our patients [14].

All patients shared a characteristic thyroid profile: elevated fT3, low fT4, and normal TSH. The fT3/fT4 ratio has been proposed as a screening tool for MCT8 deficiency, with values typically above the 97th percentile in affected individuals [15]. In our cohort, all patients had ratios exceeding 0.9. Peripheral T3 elevation is attributed to enhanced deiodination of T4 by Type 1 deiodinase in the liver and kidneys, leading to peripheral hyperthyroidism. Although some reported cases describe tachycardia and low body weight, only the latter was observed in our patients [14, 16]. Low creatinine and elevated creatine kinase levels may reflect generalized muscle hypoplasia and dystrophy. In contrast to previously reported cohorts, in which overt peripheral hyperthyroidism has been frequently described, our patients exhibited only mild thyrotoxic features such as low body weight and mild tachycardia.

The T3 analogue Triac (3,3 ^′^,5‐triiodothyroacetic acid) crosses cell membranes independently of MCT8 and has been shown to rescue the brain phenotype in Mct8‐deficient mice and safely ameliorate peripheral thyrotoxic symptoms in patients with MCT8 deficiency [17]. However, the predefined core neurological developmental outcomes—as proposed by Wilpert et al. [18]—have not been achieved in either the published Phase 2 Triac I trial [19] or the subsequently completed Triac II trial (Egetis Therapeutics, press release, 2023; full study report pending publication). These findings underscore that although peripheral manifestations can be mitigated, effective therapies targeting central neurodevelopment remain an unmet medical need [17]. This therapeutic gap is further illustrated in our cohort: Patient P6 received Triac along with systematic rehabilitation, and although his serum TH levels normalized, no significant improvement in motor or cognitive function was observed. In the absence of curative neurodevelopmental therapies, early diagnosis remains the most actionable strategy to enable timely intervention, anticipatory guidance, and avoidance of diagnostic odysseys. Although whole‐exome sequencing facilitated early genetic diagnosis in our cohort, the condition could still be overlooked without concomitant serum TH testing. Therefore, we recommend routine thyroid function screening—particularly measurement of T4 and T3—followed by targeted or broad‐spectrum genetic testing in children presenting with unexplained developmental delay, especially when accompanied by abnormal TH profiles.

Cranial MRI commonly revealed extracerebral space widening and delayed myelination, consistent with previous reports. Recent studies in Mct8‐deficient mice suggest that impaired oligodendrocyte differentiation and structural defects contribute to hypomyelination and neuronal dysfunction, aligning with the clinical phenotype [20].

We report eight previously undefined *SLC16A2* mutations, including three de novo variants. With the exception of P8, all mutations were maternally inherited. The balanced t(X; 12) translocation in P8 is the first such mutation reported in a male AHDS patient. Unfortunately, FISH validation could not be performed due to lack of guardian consent. Phenotypic severity varied among patients: P1, P2, and P5 carried missense mutations, yet p.Gly208Arg caused more severe impairment than p.Ala150Thr. Among frameshift carriers (P4, P6, and P7), p.Ser243Cysfs was associated with the mildest phenotype. Interestingly, P1 and P2—both with p.Ala150Thr—had similar initial presentations, but P1 showed better motor and cognitive outcomes after systematic rehabilitation, suggesting that early intervention may improve functional trajectories.

Functional studies indicated that mutations impair MCT8 function through multiple mechanisms, including reduced expression and defective membrane trafficking [21]. Both p.Ala150Thr and p.Ile328_Ala329delinsThr mutants showed decreased expression and increased cytoplasmic retention. Structural modeling suggested that altered helix formation, side‐chain interactions, and hydrogen bonding contribute to functional deficits. Mutant proteins that reach the plasma membrane may retain partial function, potentially explaining milder phenotypes in some cases. Further studies are needed to fully elucidate the pathogenic mechanisms of these structural changes.

## 5. Conclusion

This study reports 10 Chinese pediatric patients with SLC16A2 variants, expanding the genetic and phenotypic spectrum of AHDS. The fT3/fT4 ratio is a useful screening tool in children with developmental delay, and early WES can facilitate timely diagnosis. TH screening during the fetal and neonatal periods may enable earlier detection of the disease, facilitating early intervention and improved prognosis. Combined Triac treatment and systematic rehabilitation may offer clinical benefit, and comprehensive care is essential to reduce complications and improve quality of life. Although AHDS is clearly caused by MCT8 deficiency, further functional studies are needed to unravel the molecular mechanisms and improve early diagnosis, treatment, and genetic counseling.

## Author Contributions

Wei Li, Zijia Sun, and Xiao Wu contributed equally to this work. The first listed made the greatest contribution to the paper.

## Funding

This study was supported by the National Natural Science Foundation of the China Youth Fund (81401864).

## Conflicts of Interest

The authors declare no conflicts of interest.

## Data Availability

The data that support the findings of this study are available from the corresponding authors upon reasonable request.
